# Long-term results of thumb carpometacarpal joint arthrodesis

**DOI:** 10.1007/s00402-026-06375-4

**Published:** 2026-06-13

**Authors:** Pascal Vogler, Stefan Benedikt, Simone Bode, Alexander Keiler, Kerstin Stock, Rohit Arora

**Affiliations:** Department of Orthopaedics and Traumatology, University Hospital Innsbruck, Innsbruck, Austria

**Keywords:** Thumb carpometacarpal arthrodesis, CMC 1 joint arthrodesis, Long-term outcomes, Non-union, Patient-reported outcome measures, Grip strength restoration

## Abstract

**Introduction:**

Thumb carpometacarpal (CMC 1) arthrodesis remains an established treatment option for patients with high functional demands and strength requirements. Long-term data on mobility, complication rates and clinical outcomes remain limited. This study aimed to analyze long-term clinical and radiological results after CMC 1 arthrodesis.

**Materials and methods:**

A single-center, retrospective study included patients who underwent CMC 1 arthrodesis between 2004 and 2024. Following medical record analysis, eligible patients were invited to a standardized clinical and radiological follow-up examination. Outcomes included DASH, PRWHE and MHQ scores, pain intensity, mobility, strength and patient satisfaction. Radiological assessment covered fusion signs, implant position and adjacent joint arthrosis. The median follow-up was 9 years.

**Results:**

34 patients were included, 16 participated in follow-up. The median DASH score was 14 points, PRWHE 4 points and MHQ 80 (operated) vs. 84 (contralateral). Grip strength reached 85% of the contralateral side, with preserved pinch strength. Compensatory adaptation of adjacent joints was observed regarding range of motion. Patients reported no relevant resting pain and only minimal load-related discomfort. 15 of 16 patients were satisfied with the outcome. Non-union occurred in 4 of 34 cases (11.8%), associated with absent cancellous bone grafting or compression screws. Revision surgery was required in 3 patients, achieving bone consolidation and pain remission in all cases. Radiological follow-up revealed STT arthrosis in 27% of cases, without clinical relevance.

**Conclusion:**

CMC 1 arthrodesis demonstrates good long-term results with significant pain relief and high patient satisfaction. Mobility compensation and near-complete strength restoration were observed. Adjacent joint arthrosis occurred but remained largely asymptomatic. The use of cancellous bone grafting and compression screws is strongly recommended to reduce non-union risk. Arthrodesis proves a reliable surgical option, particularly for patients with high strength requirements.

## Introduction

Osteoarthritis of the first carpometacarpal joint is one of the most common degenerative pathologies of the hand [1]. The CMC 1 joint is also the most commonly treated joint for osteoarthritis in the hand [2]. The annual incidence is approximately 1,5% for women and 0,7% for men [3]. This high incidence can be explained by the unique anatomy of the trapezoid-metacarpal joint. Although this joint morphology allows for multiplanar mobility, it is exposed to high shear forces and quickly leads to micro instabilities [4–6]. Conservative treatment approaches, such as use of orthesis, manual therapy, analgesics and cortisol injections are particularly effective in the early stage of the disease [7–9]. In advanced stages however (usually from Eaton-Littler Stage II onwards) and with corresponding symptoms, surgical treatment is necessary [10–12]. The most common and best studied form of therapy is trapeziectomy. It is performed either as a standalone procedure or in combination with ligament reconstruction and tendon interposition (LRTI) [12]. This option is associated with a significant reduction in pain and improvement in function [2, 12–14]. Joint replacement is currently the most advanced surgical treatment for CMC1 osteoarthritis. It is indicated for patients who want rapid rehabilitation and have high functional demands. Total arthroplasty shows better function and pain reduction after 5 years, but the durability of the corresponding implants has not yet been sufficient clarified [15]. A minimally invasive arthroscopic procedure such as partial trapeziectomy with or without tendon suspension and internal stabilization has shown good results. It can be considered if the scapho-trapezial joint surface is intact [16]. Minimally invasive techniques, such as selective denervations show rapid improvements in symptoms, but their indications are limited and there are hardly any long-term results available at this point [17, 18].

Thumb carpometacarpal arthrodesis is a treatment option that is particularly suitable for patients who require high levels of strength, such as manual laborers [19] and patients who require significant strength in their thumbs due to their high level of athletic activity, such as climbers, who prioritize stability over fine motor control.

This surgical procedure shows similar patient satisfaction compared to comparable procedures for the treatment of rhizarthrosis [20, 21]. Furthermore, this procedure significantly improves pain relief in particular [19]. Another advantage of this procedure is the permanent stabilization of the joint. In addition, the arthrodesis procedure does not result in proximalization, as it can occur in trapeziectomy [20, 22]. Restricted mobility of the carpometacarpal joint due to arthrodesis is usually compensated by the adjacent joints, allowing partial compensation of impaired thumb opposition [23, 24].

Nevertheless, arthrodesis has been reported in the literature to be associated with higher complication rates. In particular, the development of non-unions has been described with incidence ranging from 8% to 21% [13, 19]. Other reported complications include adjacent joint arthritis, with scaphotrapeziotrapezoidal (STT) arthritis being of relevance [20, 22]. Achievement of osseous fusion in arthrodesis can be accomplished using various surgical techniques. Plate osteosynthesis is the most employed method and is typically combined with autologous bone grafting. Follow up studies have demonstrated high rates of mechanical stability and successful bony union with this technique compared with other methods [22, 25]. The reliability of dorsal plate osteosynthesis in thumb joint arthrodesis has also been demonstrated for the metacarpophalangeal joint, further underscoring the versatility of this fixation technique for all thumb joints [26]. Before the introduction of plate fixation systems, arthrodesis was frequently performed using tension band osteosynthesis with K-wires, however this technique has become less prominent in recent years [25]. A novel approach is screw osteosynthesis with headless compression screws [25, 27]. This procedure can also be performed with arthroscopic assistance and is therefore minimally invasive [23].

Literature provides only limited evidence on long term outcome following thumb carpometacarpal arthrodesis, with mean postoperative clinical and radiological follow-up periods ranging from 2 to 6 years [20, 22, 23, 28]. Our study presents a comprehensive long-term clinical and radiological analysis of first carpometacarpal arthrodesis over a substantially longer follow-up period than reported in comparable studies with a median follow-up of 9 years.

## Methods

The study was designed as a monocentric study covering a period of 20 years from January 1st 2004 to January 1st 2024. Approval to conduct this follow-up study was obtained from the local ethical review board. Potential cases were identified through a systematic analysis of the hospital information system using a keyword search. All retrieved records were screened for eligibility based on predefined inclusion and exclusion criteria established prior to data extraction.

Indications for arthrodesis of CMC 1 were manual workers with high functional demand such as carpenter or sportive active patients with the need of maximum stability and strength such as rock climbers.

Inclusion criteria were all patients of any age over 18 years, who have been diagnosed with thumb carpometacarpal osteoarthritis and have undergone surgical treatment involving arthrodesis of the CMC 1. Another inclusion criteria was the availability of a surgical report and the surgery was performed at least 1 year ago. Patients were excluded if they declined their participation in the study, if no operative report was available or the surgical procedure had been performed at an external institution. Another reason for exclusion was, if the clinical examination and standardized questioning could not be reliably conducted. This included individuals with comorbidities such as severe neurological disorders or dementia, which could compromise the validity of the clinical assessments.

For all included patients identified through the search, a complete medical history was reviewed. The data extraction followed a standardized procedure to minimize bias and ensure consistent documentation across the evaluation. The following parameters were extracted from the medical records and surgical documentation. The demographic information included sex, date of birth, side dominance, smoker or non-smoker, affected side, age, when the surgery was performed. Regarding the preoperative radiological data, the degree of osteoarthritis (Eaton and Littler Score) was extracted from the records. According to this classification, osteoarthritis of the CMC 1 joint is divided into 4 stages. Stage I describes a slight reduction in joint space, minimal osteophytes without subluxation. Stage II describes a significant reduction in joint space. Osteophytes less than 2 mm and incipient subchondral sclerosis without, or only minimal subluxation. Stage III describes further narrowing of the joint, larger osteophytes greater than 2 mm, subchondral sclerosis and cysts, as well as possible subluxation of the joint. The final stage IV describes advanced osteoarthritis with joint destruction and sclerosis, also involving adjacent joints such as the STT (Scapho-trapezio-trapezoidal) joint, as well as pronounced subluxations or luxation [29], although the correlation between radiological staging and intraoperative cartilage findings has shown limitations [30]. The surgical parameters included the date of surgery and the surgical technique. Regarding the postoperative information, the length of hospital stay, all documented complications, such as implant loosening, failure, non-union, infection, complex regional pain syndrome, vascular or nerve injuries, tendon injuries and any revision procedures were assessed.

All patients included in the cohort were invited to attend a follow-up examination at our institution. In the case of agreement, written informed consent was obtained. During this visit, a standardized clinical assessment was performed. First, the patients were interviewed, using a questionnaire. During the interview, outcome scores were collected including the German version Disabilities of the Arm, Shoulder and Hand (DASH) score [31], the German version Patient-Rated Wrist/Hand Evaluation (PRWHE) [32] and the German version of Michigan Hand Outcomes Questionnaire (MHQ) [33]. The Patients were asked to rate their pain level using a Numeric Rating Scale (NRS) in comparison with the contralateral side (0 equals no pain, 10 equals severe pain). The patients were also questioned about whether they would choose to undergo the surgery again and were asked to indicate their satisfaction level (not satisfied, satisfied and very satisfied). Following the interview, a clinical test was performed. This testing was always in comparison with the contralateral side. The range of motion of the MCP and IP joint was measured, using a goniometer. Opposition in the thumb were measured via the Kapandji score. Subsequently strength testing was performed including measurements of grip strength, key pinch, tip pinch (D1/D2) and three-jaw pinch, using a calibrated hand dynamometer (Baseline^®^ Hydraulic Hand Dynamometer - Fabrication Enterprises Incorporated (FEI) - PO Box 1500, White Plains, NY 10602 (USA)). For the radiological follow-up analysis, a standardized X-ray of the CMC 1 joint was performed in two planes. This allowed the implant position, bone fusion of the arthrodesis, possible adjacent arthrosis to be analyzed. STT arthrosis was graded after Crosby et al. from Grade 0 (no abnormality), Grade 1 (narrowing half of the normal joint space), Grade 2 (barely defined joint space) and Grade 3 (Erosion, sclerosis and cystic changes) [34]. From Grade 1 onwards, the finding was classified as STT osteoarthritis. Detailed literature research was performed via PubMed^®^ and Google Scholar^®^. The scientific search platform Open Evidence^®^ was used for supplementary literature research.

## Results

The analysis identified a total of 34 patients who had undergone a thumb carpometacarpal joint arthrodesis due to osteoarthritis. Those cases were retrospectively assessed regarding demographic data, surgical technique, preoperative status, perioperative course, complications and revision procedures. Of these patients, 16 (47,1%) were able to attend the clinical and radiological follow-up examination. The average follow-up period across the 16 patiens was on median 9 years (P^25^: 5; P^75^: 14) ranging from 1 to 20 years.

### Demographic data and operated side

The entire study population consisted of 21 women (61,8%) and 13 men (38,2%). In 14 cases (41,2%), the left hand was operated on, while 20 patients (58,8%) underwent arthrodesis on the right side. The median age at the time of surgery was 54 years (P^25^: 49; P^75^: 58). The median age during the follow-up examination of the 16 patients was 66 years (P^25^: 56; P^75^: 67). In this follow up group, the dominant side was operated in 12 of 16 cases (75%).

### Indication for surgery

The indication for surgery was determined according to the Eaton and Littler classification in symptomatic patients. Two patients (5,9%) in stage I, 16 (47,1%) in stage II, 15 (44,1%) in stage III and one patient (2,9%) in stage IV.

### Surgical techniques and perioperative course

**Fig. 1:**
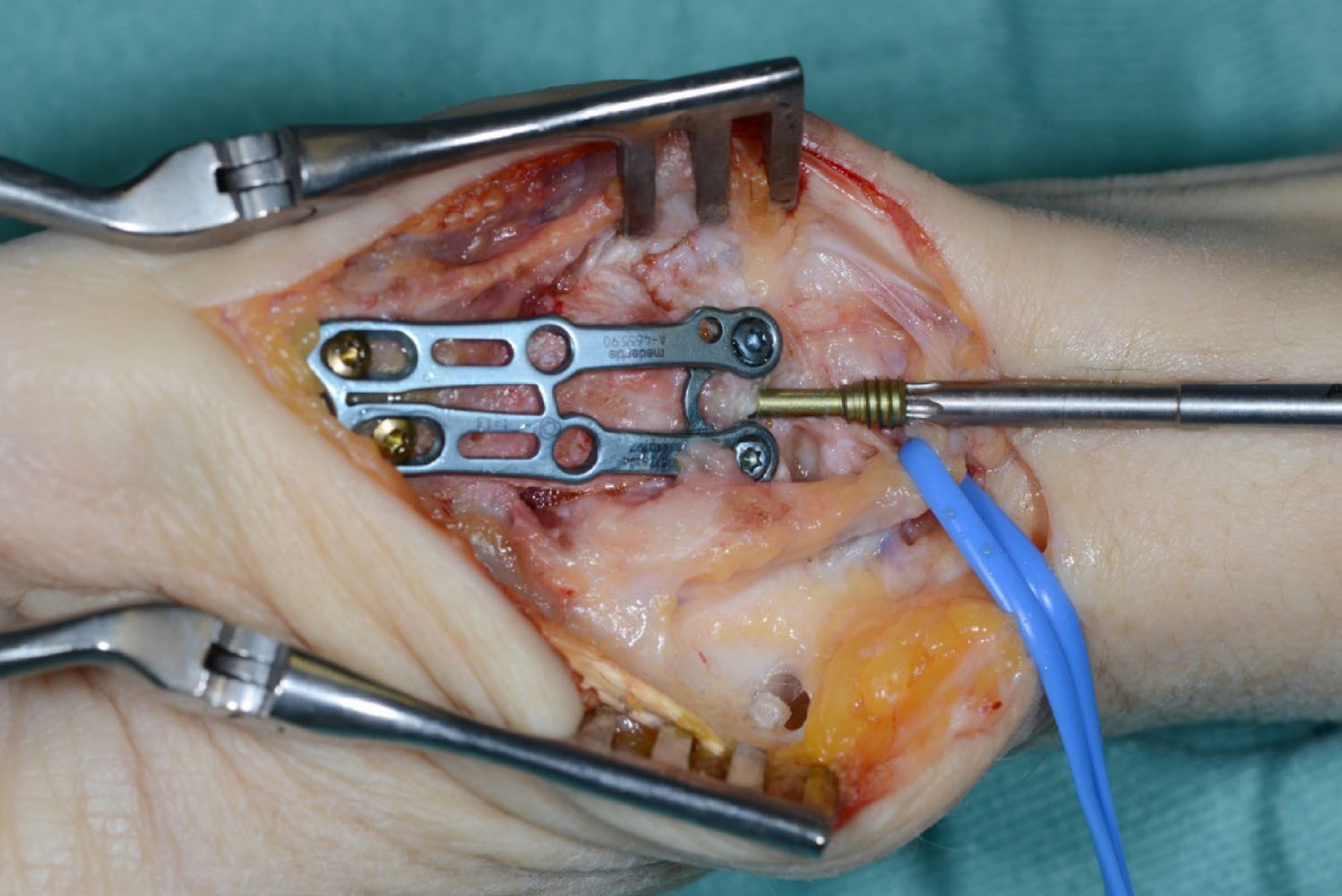
Perioperative image showing use of the CMC-I fusion system 2.0, 3.0 (Medartis AG, Hochbergerstrasse 60E, 4057 Basel, Switzerland), an angular stable plate in combination with a 3.0 headless compression screw

**Fig. 2:**
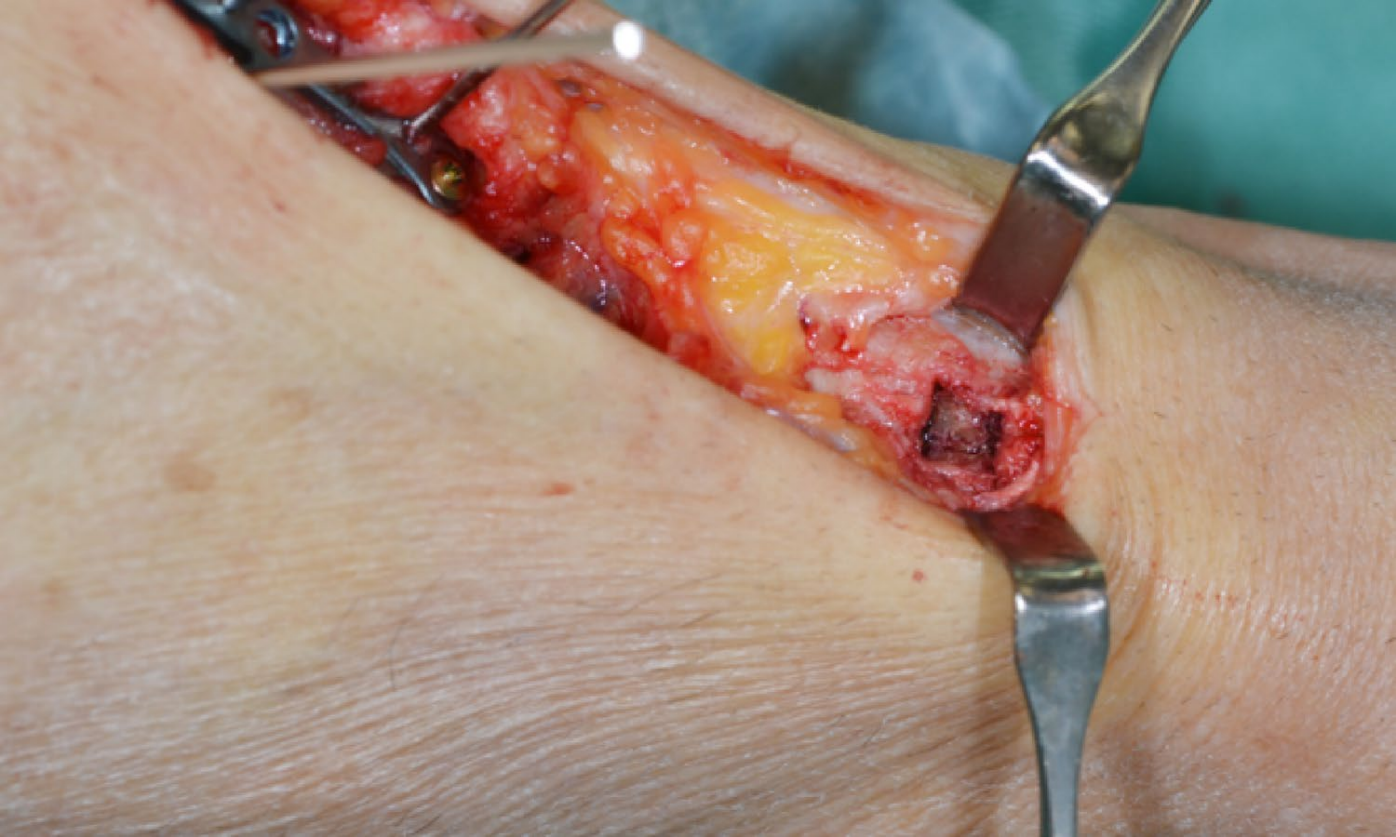
Harvesting of a distal radial cancellous bone graft between the first and the second extensor compartment via single access

With 24 cases (70,6%), plate osteosynthesis (example see Figs. 1 and 2) was the most common technique for arthrodesis. Table 1 provides an overview of the procedures and the additional use of bone grafting. The mean length of hospital stay was 2 days (P^25^: 2; P^75^: 3). The median postoperative immobilization period was 5 weeks (P^25^: 4; P^75^: 5).

**Table 1:** Surgical techniques

Surgical technique	Number	Bone grafting
Plate arthrodesis	24	13
Tension-band wiring	7	2
Plate arthrodesis combined with K-wire fixation	2	1
K-wire fixation	1	0

### Complications and revision procedures

**Fig. 3:**
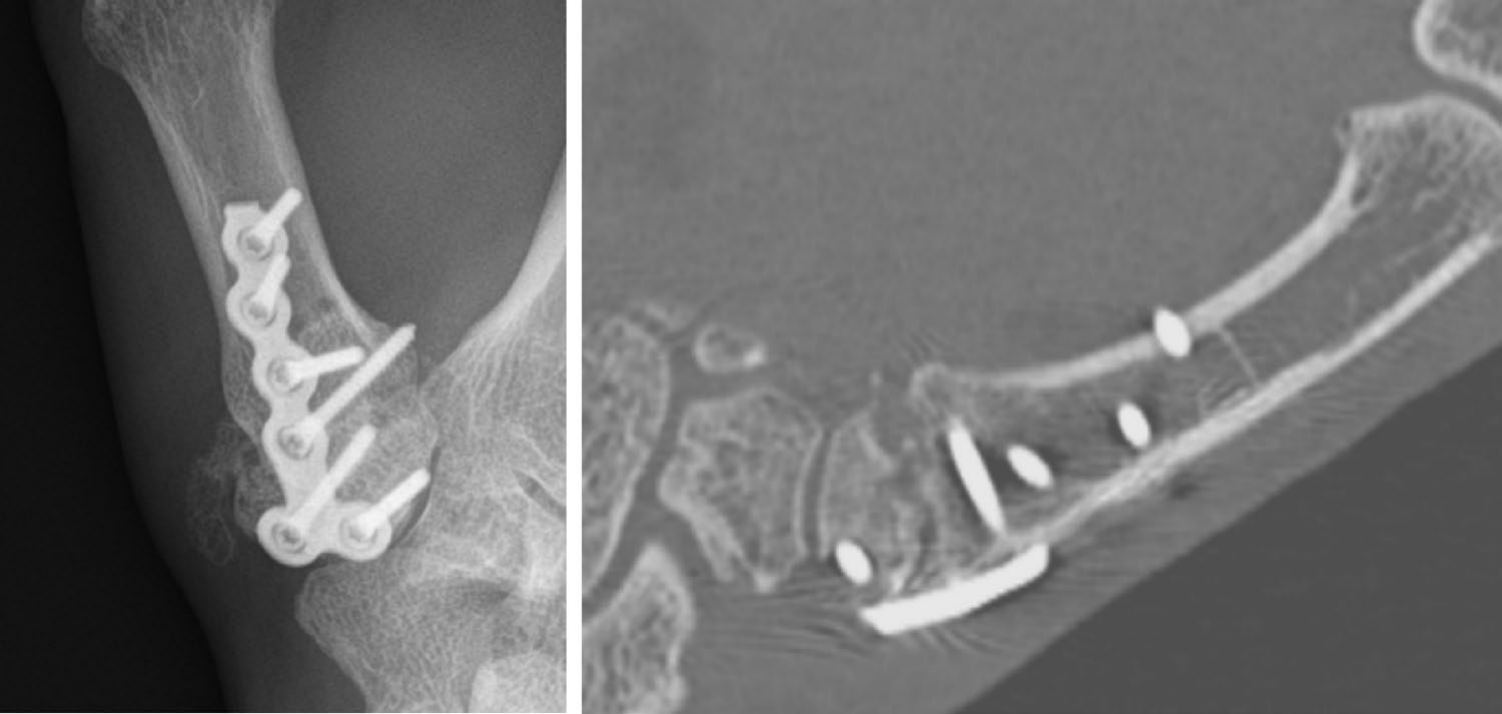
Non-union after arthrodesis using plate fixation without compression screws and without additional bone graft

A total of five major complications were identified, which results in a rate of 14,7% of all arthrodesis patients examined. Four of the five major complications (11,8% of all patients) were non-unions. Three of these cases were surgically treated by hardware removal and re-arthrodesis with a cancellous bone graft. In one case with moderate symptoms, no surgical intervention was necessary. All four non-unions occurred in procedures with plate arthrodesis (different systems). Three of the four cases were performed without bone grafting. Two of the four patients underwent plate fixation without compression screws (Fig. 3). In one patient, rupture of the flexor pollicis longus tendon occurred due to a palmarly protruding k-wire. In one case, plate breakage occurred as a minor implant-related complication. Despite this, osseous healing was achieved and the patient was free of symptoms after implant removal. Further hardware removals were performed due to skin irritation over the implant in 9 patients (26,5%).

### The following results are from the 16 patients who underwent follow-up examinations

#### Clinical assessment in follow up cohort

Tables 2 and 3 show the clinical measurements, in comparison to the contralateral side. Table 4 shows the subjective patient satisfaction. One patient (6,3%) reported dissatisfaction. The results of the clinical scores are listed in Table 5.

**Table 2:** Functional outcomes: Kapandji score and range of motion in follow up cohort (*n* = 16, values presented as medians with 25% and 75% percentile)

	Operated side	Contralateral side	% Range of motion compared to contralateral side
Kapandji- score	9 [P_25_: 8; P_75_:10]	10 [P_25_:9; P_75_:10]	
IP joint (flexion in degrees)	70 [P_25_: 60; P_75_: 74]	70[P_25_: 60; P_75_: 85]	100%
MCP joint (flexion in degrees)	55 [P_25_: 45; P_75_: 62]	55 [P_25_: 40; P_75_: 60]	100%

**Table 3:** Grip strength in follow-up cohort (*n* = 16, values presented as medians with 25% and 75% percentile)

Method	Operated side (kg)	Contralateral side (kg)	% Strength compared to contralateral side
Grip strength	26 [P_25_: 22; P_75_: 33]	31 [P_25_: 21; P_75_: 40]	84%
Key pinch	6 [P_25_: 5; P_75_: 9]	6 [P_25_: 6; P_75_: 8]	100%
Tip pinch (D1 / D2)	4 [P_25_: 3; P_75_: 5]	4 [P_25_: 3; P_75_: 6]	100%
Three-jaw chuck pinch	5 [P_25_: 5; P_75_: 7]	6 [P_25_: 5; P_75_: 8]	83%

**Table 4:** Patient satisfaction and pain assessment (*n* = 16, metric values presented as medians with 25% and 75% percentile)

Outcome measure	Result
NRS pain score – at rest	
Operated side	0 [P_25_: 0; P_75_: 1]
Contralateral side	0 [P_25_: 0; P_75_: 1]
NRS pain score – during activity	
Operated side	1 [P_25_: 0; P_75_: 4]
Contralateral side	0 [P_25_: 0; P_75_: 3]
Would you undergo the operation again?	
Yes/No	12/4
Overall satisfaction with surgical outcome	
Very satisfied	11
Satisfied	4
Not satisfied	1

**Table 5:** Outcome score values in follow-up cohort (*n* = 16, values presented as medians with 25% and 75% percentile)

Score	Value (points)
DASH - disabilities of the arm, shoulder and hand	14 [P_25_: 1; P_75_: 23]
PRWHE - patient-rated wrist/hand evaluation	4 [P_25_: 1; P_75_: 33]
MHQ - Michigan Hand Outcome Questionnaire	Operated side: 80 [P_25_: 56; P_75_: 89] Contralateral side: 84 [P_25_: 63; P_75_: 98]

#### Radiological assessment in follow up cohort

**Fig. 4:**
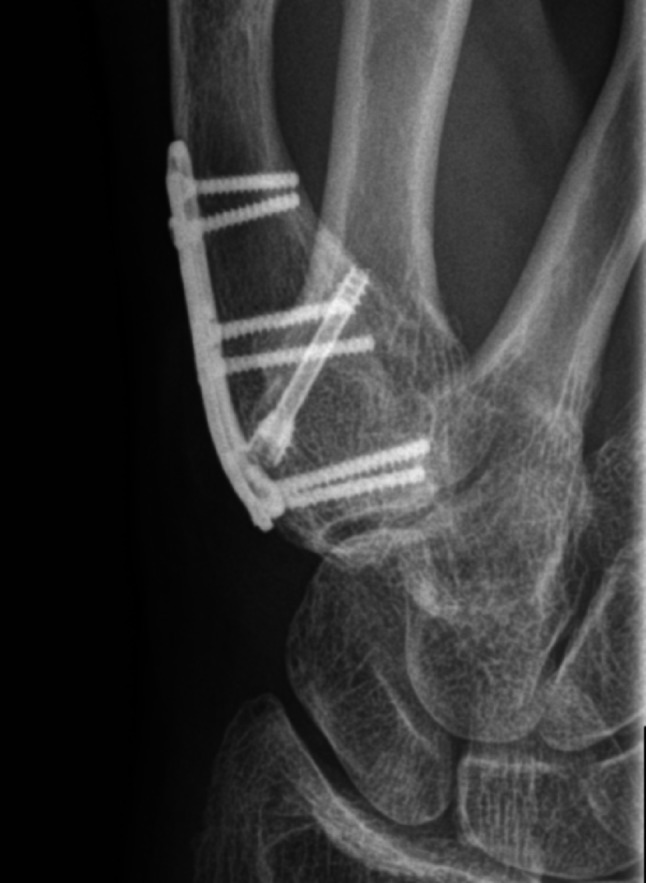
Sufficient bony fusion after angular-stable plate arthrodesis with bone graft and headless compression screw

**Fig. 5:**
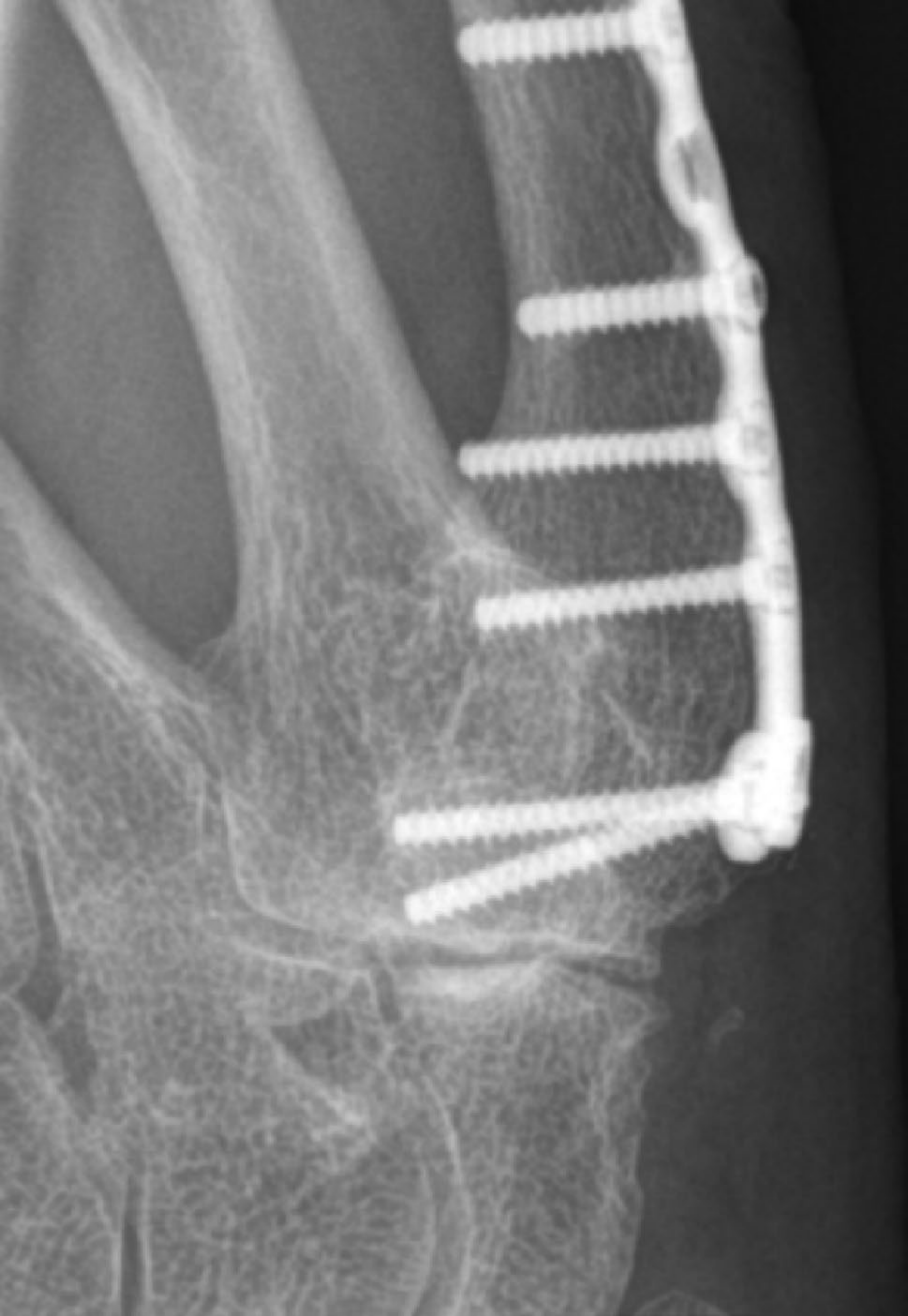
Example for adjacent joint osteoarthritis involving the STT joint after CMC 1 plate arthrodesis

Of the 16 patients, 15 underwent radiological follow-up using conventional x-rays. One patient refused to have an X-ray taken. Bony fusion of the arthrodesis was observed in all patients examined (Fig. 4). Implant failure of dislocation was not observed in any of the cases. Adjacent joint degeneration in the form of scaphotrapeziotrapezoidal osteoarthritis was observed in 5 of 15 patients (26,7%) (Fig. 5).

Tables 6 and 7 provide a comparison of the follow-up group between developed adjacent osteoarthritis and without adjacent osteoarthritis in terms of both outcome scores and patient satisfaction.

**Table 6:** Patient satisfaction by STT osteoarthritis status

Satisfaction	STT OA (*n* = 4)	No STT OA (*n* = 11)
Very satisfied	2 (50,0%)	1 (9,1%)
Satisfied	2 (50,0%)	9 (81,8%)
Not satisfied	0 (0%)	1 (9,1%)
Satisfied/Very satisfied	4 (100%)	10 (90,9%)

**Table 7:** Outcome scores and NRS for pain by STT osteoarthritis status (values presented as medians with 25% and 75% percentile)

Outcome parameter	STT OA (*n* = 4)	No STT OA (*n* = 11)
NRS	At rest: 1 [P_25_: 0; P_75_: 2] During activity: 2 [P_25_: 0; P_75_: 4]	At rest: 0 [P_25_: 0; P_75_: 0] During activity: 1 [P_25_: 0; P_75_: 5]
DASH	14 [P_25_: 2; P_75_: 58]	16 [P_25_: 1; P_75_: 23]
PRWHE	12 [P_25_: 2; P_75_: 59]	2 [P_25_: 1; P_75_: 37]
MHQ	Operated side: 65 [P_25_: 46; P_75_: 93] Contralateral side: 64 [P_25_: 45; P_75_: 82]	Operated side: 82 [P_25_: 55; P_75_: 90] Contralateral side: 86 [P_25_: 64; P_75_: 99]

## Discussion

The aim of this study was to conduct a sufficient long-term follow-up examination of thumb carpometacarpal joint arthrodesis performed at our department. Of the patients contacted, almost half were able to attend a clinical and radiological follow-up examination. This high number provides a good overview of the long-term results with a median follow-up period of 9 years ranging from two to 20 years after surgery.

With reference to epidemiology and gender distribution, similar results can be seen as in earlier publications, such as the comparable study by Rizzo et al. (2009), in which women were significantly overrepresented with 61,8% [35]. The average age was 53,8 years. Comparing this to other studies, our patient population appears to be slightly younger, with comparable studies showing an average age at surgery in the sixth decade of life [22, 35]. This result also correlates with the recommendation to use this procedure in more younger patients [19, 20, 36].

The most common method of fixation in our population was the use of a locking plate with the addition of autogenous bone graft and a compression screw as shown in Figs. 1 and 2. This method is also described in the literature as the preferred procedure for achieving high stability and fusion rates and for reducing the complication rate [22, 23, 25, 27]. The findings of Harenberg et al. show comparable results in 77 patients who underwent compression plate arthrodesis for osteoarthritis of the first metacarpophalangeal joint with a mean follow-up period of 7.8 years. Similar rates were observed regarding restoration of grip strength, patient satisfaction, and complication rate [37]. With a complication rate of 14,7% and a non-union rate of 11,7% the results observed in our cohort is consistent with previously published comparable data (8–25%) and compares favorably with reported rates [13, 20, 23, 25, 38]. Notably, cancellous bone grafting was omitted in three of four non-union cases. One patient with non-union presented with only mild symptoms and needed no further surgical treatment. In line with previous publications, this finding supports the recommendation to routinely use cancellous bone grafting [22, 25]. Notably, all surgically treated non-unions demonstrated radiographic consolidation at follow up. In addition to the non-union, there was another major complication in which a protruding K-wire ruptured the flexor pollicis tendon, which was treated with metal removal and tendon reconstruction. A minor complication was also observed, whereby a plate break was detected in a patient with a bone union and implant removal was indicated, but no further measures were necessary. No further complications such as described by the comparable follow up by Rizzo et al. as infections, damage to the radial nerve and its branches, intraoperative fractures of complex regional pain syndrome [35] were found in our follow-up examination.

Overall, the present results show satisfying long-term clinical outcome after thumb carpometacarpal joint arthrodesis. Several frequently raised criticisms of this treatment option could be dismissed based on objective measurement data. A key argument against carpometacarpal joint arthrodesis of the thumb is the restriction of thumb mobility [19, 20, 39]. Based on our results, this assumption cannot be confirmed. As shown in Table 2, the Kapandji scores for evaluating opposition in the medians showed almost identical values between operated and contralateral, non-operated sides (median Kapandji score 9 vs. 10). The mobility of the interphalangeal joint (IP-joint) and the metacarpophalangeal joint (MCP-joint) were also comparable on both sides, with no evidence of functional limitations after arthrodesis. This result suggests a compensatory adjustment of thumb mobility, in which the lack of mobility in the CMC joint might be functionally compensated by increased mobility in the STT and MCP joint. This shows that the overall mobility of the thumb after CMC arthrodesis is not substantially restricted, but is rather maintained through adaptive mechanisms. The 2018 study by Dormitorio et al. proves this theory of compensatory hypermobility adjacent joints after arthrodesis in their radiological comparative study [40]. The frequently cited argument of a significant restriction of functional mobility after this treatment option must therefore be viewed critically based on our data. Although there is a restriction of movement, this is partly compensated for by adjacent joints.

Very encouraging results were also seen in terms of strength recovery. Table 3 documents good clinical outcomes. This is particularly relevant as strength and stability are among the main arguments for choosing arthrodesis. The average loss of grip strength compared to the contralateral side was only about 16%, which can be classified as clinically moderate. In addition, there was no relevant difference in strength between operated and non-operated sides in the key grip and tip pinch tests. These findings emphasize that arthrodesis enables stability in force transmissions and ensures good functional resilience in the long term. The significant benefit of preserving muscle strength after this surgical method has already been proven in numerous publications [19, 20, 22, 23, 27, 28].

The patient-reported outcome measures (PROMs) collected indicate favorable functional outcomes after thumb carpometacarpal joint arthrodesis overall. Both the DASH and PRWE scores were in the low range, indicating minimal functional impairment in everyday life and a satisfactory pain-related outcome. These findings suggest that arthrodesis of the CMC 1 joint enables functionally relevant use of the hand in everyday life despite permanent joint stiffness. The MHQ allows for a differential assessment of hand function, taking into account the operated and contralateral sides. With a mean MHQ score of 80 points for the operated hand and 84 points for the contralateral hand, there was only a slight difference between the sides. These results can be interpreted as functionally good and indicate that the subjectively perceived hand function after CMC 1 arthrodesis is only slightly impaired in the long term. The small difference between the two sides suggests that surgical stiffening of this joint does not cause relevant restriction of overall hand function.

The mean DASH score was 14 points, which is a good to very good result. Due to the frequent use of this score, it can be easily compared with other surgical procedures. For resection arthroplasty, Tosun et al. report a DASH score of 13.8 in a 2,5 year follow up study [41], while Klim et al. obtained a score of 18, 12 months postoperatively [42]. Compared to the results of Davis et al., who obtained a significantly more moderate patient-reported outcome score with their 1-year DASH score of 37 [43], we can highlight significantly more satisfactory results with our results in terms of the DASH score in results of resection arthroplasty. DASH scores were also collected postoperatively after implantation of thumb carpometacarpal prostheses. Froschauer et al. reported a DASH score of 12 after a one year of follow-up [44], while Jurča et al. reported a score of 22 three years postoperatively [45]. In a recent retrospective study of 2025, Frey et al. examined 78 patients (88 prostheses) who had been treated with the TOUCH^®^ Dual Mobility prosthesis, with a mean follow-up period of 24 months. The QuickDASH score improved significantly from a mean of 51 preoperatively to 30 postoperatively after 24 months [46]. When comparing these results with our arthrodesis cohort, the postoperative DASH score in our study (median 14 points) compares favorably with the QuickDASH score reported after prosthesis replacement, despite the significantly longer follow-up period of 9 years. The meta-analysis by Kim et al. facilitates comparison of our findings with previously published follow-up outcomes after thumb carpometacarpal arthrodesis. In their study, the DASH scores ranged between 11 and 15 [19]. Komura et al. reported a DASH score of 12.4 after 20 months [23], and Hayashi et al. reported a score of 11.1 after 2–5 years [22]. In summary, our DASH scores are in line with previous DASH scores reported following arthrodesis. Compared to other methods, our results are better for resection arthroplasty; for prosthetics, however, the picture is mixed: some publications report a better DASH score, while in another, the QuickDASH score for the endoprosthesis is worse than that for arthrodesis. However, the follow-up periods were significantly shorter, which makes it difficult to compare our results with those of other studies and highlights that long-term results are crucial for measuring actual patient outcomes.

Our mean PRWE was 4 points, indicating low pain levels and low functional limitations. Compared to resection arthroplasty, the follow-up study by Klim et al. showed a PRWE of 18 points after a postoperative period of 15 months [42]. This suggests that arthrodesis is not inferior to alternative procedures in terms of pain-related and functional patient-reported outcomes. Nevertheless, the different follow-up periods must also be taken into account in this comparison.

The MHQ offers a valid method for assessing hand function [47]. In our study, the mean score for the operated side was 80, compared to 84 for the contralateral hand. For comparison, Chang et al. reported an MHQ of 67 for the operated side after resection arthroplasty. Their study did not include a comparison with the contralateral side [48]. Normal values in the healthy population are reported in the literature as approximately 95 points [49]. This shows that our results indicate an approximation to near-normal subjective hand function despite previous surgical treatment.

Nevertheless, it must be noted that age can naturally influence the outcome scores for all three surgical techniques, as the various methods have different age focuses.

The objectively good functional results and favorable PROM values are consequently also reflected in high patient satisfaction. At rest, there were no differences in pain perception between operated and contralateral sides. During activity, patients reported only minor pain, averaging 1/10 on the numerical rating scale. It is particularly noteworthy that 75% would choose this surgical treatment again, which impressively confirms the subjectively high acceptance and satisfaction with the treatment outcome.

A relevant proportion of 26,7% of the patients examined during follow-up developed adjacent joint osteoarthritis. All patients with described STT arthrosis described good to very good satisfaction levels. None of the patients diagnosed with STT osteoarthritis required further surgery. The outcome scores present a mixed picture. On the one side, patients with STT-OA show lower satisfaction in the PRWHE score, but higher satisfaction in the DASH score. The MHQ score also shows a slight difference between the STT-OA group and the group without STT-OA, to the disadvantage of the STT osteoarthritis group. However, there is no difference between the operated and contralateral sides in the STT-OA group regarding MHQ score. Due to the low sample size in the STT osteoarthritis group, no clear conclusion can be made from the data.

The main strength of this study was the long follow-up period of 9 years (median) and a maximum observation period of 20 years. Long-term follow-up data are only available to a limited extent in literature and therefore make a relevant contribution to the assessment of the sustainability of this surgical procedure. It also provides corresponding clinical, functional, and radiological results. In addition, the combination of standardized clinical tests and patient-reported outcome measures, as well as radiological follow-up examinations, provides a robust and validated basis for the multidimensional evaluation of the long-term results of this surgical method.

At the same time, however, limitations in this study must also be taken into account. The retrospective, monocentric design of this study is fundamentally vulnerable to selection and information bias. In particular, the limited number of patients who underwent clinical follow-up examinations may lead to a distortion of the results, Furthermore, no standardized follow-up examinations were performed at defined postoperative follow-up intervals, so that changes between the early postoperative phase and the long-term results could not be systematically recorded. Prospective, multicenter comparative studies would be necessary to clarify outstanding questions, in particular those concerning comparability and superiority or inferiority to other methods.

## Conclusion

The available data show promising long-term results. The often-mentioned secondary arthrosis, especially STT arthrosis, was also identified in our follow-up, but it was predominantly clinically asymptomatic and had no considerable negative impact on the overall functional outcome. Non-unions are the most common complications, especially when no bone graft is used. Compared to other surgical methods, arthrodesis shows comparable patient-reported and clinical results. Based on our findings, we propose a differentiated indication scheme for the three main surgical treatment options for osteoarthritis of the first metacarpophalangeal joint. Resection arthroplasty remains an established option with low complication rates and high patient satisfaction and may be the preferred choice for older, less active or low functional demanding patients for whom restoration of strength is of secondary importance. Treatment with an arthroplasty offers advantages in terms of early mobilization and potentially better short-term functional outcomes, making it particularly suitable for patients who prioritize range of motion and rapid rehabilitation. However, concerns regarding long-term durability and implant-related complications must be considered. As demonstrated in the present study, CMC-1 arthrodesis should primarily be recommended for younger, physically active, functional high demanding patients and those engaged in manual labor for whom grip strength and joint stability are of primary importance. Crucially, our data did not confirm the frequently cited concern regarding restricted range of motion. Due to compensatory involvement of adjacent joints (MCP and STT), median Kapandji scores and range of motion in the IP/MCP region were largely preserved during long-term follow-up. Grip strength was restored to a level similar to the contralateral side, while fully preserving the key pinch and tip pinch. Therefore, arthrodesis should not be viewed as a “last resort”, but rather as a reliable first-line option for the appropriate patient profile, accepting a potential minor compromise in mobility in exchange for long-term stability and superior preservation of strength.

## Data Availability

The datasets generated and/or analysed during the current study are not publicly available but are available from the corresponding author on reasonable request.
